# Novel Surveillance View: A Novel Benchmark and View-Optimized Framework for Pedestrian Detection from UAV Perspectives

**DOI:** 10.3390/s25030772

**Published:** 2025-01-27

**Authors:** Chenglizhao Chen, Shengran Gao, Hongjuan Pei, Ning Chen, Lei Shi, Peiying Zhang

**Affiliations:** 1 Qingdao Institute of Software, College of Computer Science and Technology, China University of Petroleum (East China), Qingdao 266580, China; chenglizhaochen@upc.edu.cn (C.C.); s22070060@s.upc.edu.cn (S.G.); zhangpeiying@upc.edu.cn (P.Z.); 2 Shandong Key Laboratory of Intelligent Oil & Gas Industrial Software, Qingdao 266580, China; 3 School of Engineering Science, University of Chinese Academy of Sciences, Beijing 100049, China; 4 School of Information and Communication Engineering, Beijing University of Posts and Telecommunications, Beijing 100876, China; nchen@bupt.edu.cn; 5 Key Laboratory of Intelligent Game, Yangtze River Delta Research Institute of NPU, Taicang 215400, China; leiky_shi@bupt.cn; 6 State Key Laboratory of Media Convergence and Communication, Communication University of China, Beijing 100024, China; 7 Key Laboratory of Education Informatization for Nationalities (Yunnan Normal University), Ministry of Education, Kunming 650092, China

**Keywords:** UAV perspective datasets, pedestrian detection, deformable convolution, feature decomposition, attention mechanism

## Abstract

To address the issues of insufficient samples, limited scene diversity, missing perspectives, and low resolution in existing UAV-based pedestrian detection datasets, this paper proposes a novel UAV-based pedestrian detection benchmark dataset named the Novel Surveillance View (NSV). This dataset encompasses diverse scenes and pedestrian information captured from multiple perspectives, and introduces an innovative data mining approach that leverages tracking and optical flow information. This approach significantly improves data acquisition efficiency while ensuring annotation quality. Furthermore, an improved pedestrian detection method is proposed to overcome the performance degradation caused by significant perspective changes in top-down UAV views. Firstly, the View-Agnostic Decomposition (VAD) module decouples features into perspective-dependent and perspective-independent branches to enhance the model’s generalization ability to perspective variations. Secondly, the Deformable Conv-BN-SiLU (DCBS) module dynamically adjusts the receptive field shape to better adapt to the geometric deformations of pedestrians. Finally, the Context-Aware Pyramid Spatial Attention (CPSA) module integrates multi-scale features with attention mechanisms to address the challenge of drastic target scale variations. The experimental results demonstrate that the proposed method improves the mean Average Precision (mAP) by 9% on the NSV dataset, thereby validating that the approach effectively enhances pedestrian detection accuracy from UAV perspectives by optimizing perspective features.

## 1. Introduction

In recent years, significant advancements in computer vision have greatly accelerated the development of object detection technologies [1,2,3], particularly in the area of pedestrian detection [4,5]. As a fundamental and critical research topic within computer vision, pedestrian detection plays an essential role in a wide range of applications, including public security surveillance [6], intelligent transportation systems [7], and autonomous driving [8]. Concurrently, the rapid growth and widespread deployment of UAV technology [9,10] have introduced new possibilities for real-time monitoring [11] and large-scale area surveillance [12]. This shift has made pedestrian detection from UAV perspectives an increasingly prominent area of research [13,14,15,16,17]. However, unlike traditional ground-based camera perspectives, UAV-based perspectives introduce extreme viewpoint distortions and occlusion challenges, significantly affecting the accuracy and stability of existing pedestrian detection models.

**Figure 1:**
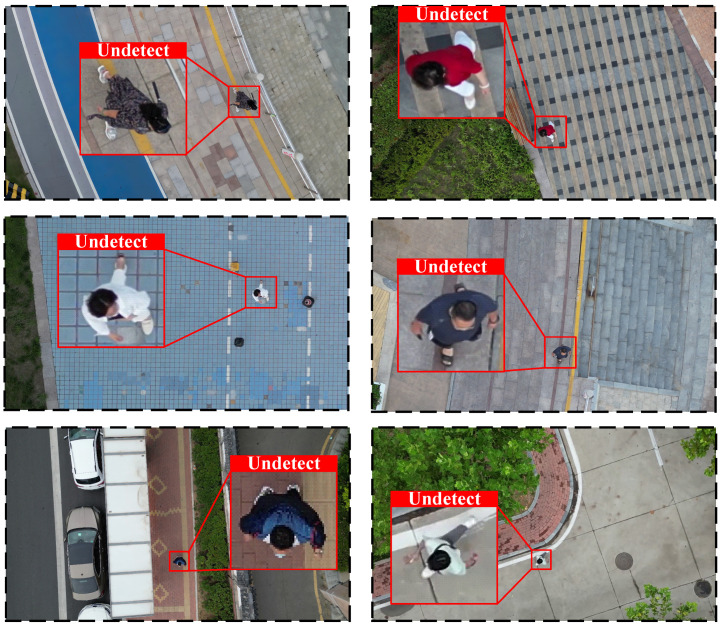
Mainstream pedestrian detectors are unable to detect pedestrians effectively from a UAV perspective.

Currently, most mainstream pedestrian detection algorithms are trained and optimized for standard perspectives, such as ground-level views. However, their performance often deteriorates significantly when applied to non-standard perspectives, such as aerial views. As a result, existing pedestrian detection models encounter considerable challenges in achieving accurate identification and precise localization in these settings. One key issue is the distortion of appearance caused by changes in perspective. In UAV overhead views, the shape and features of pedestrians differ markedly from those in traditional ground-level perspectives, making feature extraction more challenging. This discrepancy hampers the ability of conventional detection models to recognize and locate targets reliably. Furthermore, the high-altitude perspective introduces additional background interference. As the scene complexity increases, distinguishing between background elements and the target becomes progressively more difficult. Additionally, fluctuations in UAV flight altitude lead to significant variations in pedestrian size within the image, further complicating the detection process, especially when dealing with pedestrians of diverse scales. As illustrated in Figure 1, YOLOv7 [18] struggles to effectively detect pedestrians in UAV perspectives, highlighting the limitations of current algorithms in aerial settings.

These issues underscore the limitations of current pedestrian detection technologies when applied to UAV perspectives. Moreover, most existing datasets are predominantly centered around ground-level perspectives and feature relatively low resolution, which fails to address the unique requirements of pedestrian detection from UAV viewpoints. This limitation prevents the provision of fine-grained image information necessary for improving model performance. The absence of large-scale, high-quality pedestrian detection datasets specific to UAV perspectives directly impedes the effectiveness of models in real-world applications. Furthermore, traditional manual annotation is time-consuming, labor-intensive, and prone to inconsistencies and accuracy deviations, particularly when handling the complex and dynamic perspectives inherent in UAV imagery. As such, there is an urgent need to develop specialized datasets and detection methods tailored to UAV-based pedestrian detection to enhance the accuracy and robustness of models in these settings.

This paper proposes a novel method to enhance the performance of existing models in UAV-based pedestrian detection, thereby addressing the challenges associated with pedestrian detection from UAV perspectives. The method builds upon the YOLOv7 architecture and introduces three key innovations: First, the View-Agnostic Decomposition (VAD) module decouples pedestrian features into view-dependent and view-independent branches, enhancing the model’s ability to generalize across multiple perspectives. Second, the Deformable Conv-BN-SiLU (DCBS) module employs deformable convolutional techniques to dynamically adjust the receptive field shape, thereby improving the model’s adaptability to the geometric deformations of pedestrians in UAV views. Third, the Context-Aware Pyramid Spatial Attention (CPSA) module integrates multi-scale feature pyramids with spatial attention mechanisms, significantly strengthening the model’s feature extraction capability. To validate the effectiveness of the proposed approach, this paper also introduces a novel pedestrian detection dataset specifically tailored for UAV perspectives. The dataset covers a wide range of diverse scenarios, including varying viewpoint angles, crowd densities, lighting conditions, and background elements, thereby providing a more realistic simulation of real-world complexities. Furthermore, a hybrid annotation method based on tracking is proposed, which combines multi-object tracking algorithms with optical flow estimation techniques. This approach improves annotation efficiency and enhances accuracy, thereby laying a solid foundation for creating a high-quality UAV-specific pedestrian detection dataset, NSV, which will serve as a reliable resource for subsequent algorithm development and performance evaluation. The main contributions of this work are as follows:

1. We introduce the Tracking-based Automatic Hybrid Annotation (TAHA) method, which leverages the complementary strengths of tracking and optical flow techniques in conjunction with inter-frame motion characteristics to achieve precise and efficient automatic labeling of video frames. This approach effectively overcomes the time-consuming and labor-intensive challenges associated with traditional manual annotation, significantly enhancing both the efficiency and accuracy of the annotation process.
2. We propose NSV, a novel UAV-based pedestrian detection dataset encompassing diverse scenes, multiple viewpoint angles, and multi-scale pedestrian annotations. NSV effectively addresses common challenges in UAV-based pedestrian detection, including insufficient sample sizes, limited scene diversity, missing viewpoints, and low resolution. By overcoming these issues, NSV provides a robust foundation for enhancing the performance of pedestrian detection models from UAV perspectives.
3. We develop Pedestrian-DVC, a pedestrian detection model designed explicitly for UAV perspectives. This model effectively addresses performance degradation resulting from severe viewpoint changes, significant scale variations, and complex background interference by integrating the VAD, DCBS, and CPSA modules. By utilizing these modules, Pedestrian-DVC significantly enhances pedestrian detection accuracy in UAV-based scenarios.

## 2. Related Work

### 2.1. UAV Datasets

In recent years, the rapid advancement in UAV technology and its widespread applications across various domains have led to the emergence of several datasets designed explicitly for pedestrian detection from UAV perspectives, including VisDrone2019 [19] and Okutama-Action [20]. These datasets provide rich data support for pedestrian detection from UAV perspectives and drive the research and development of pedestrian detection algorithms in diverse application scenarios.

The VisDrone2019 dataset, developed by the Machine Learning and Data Mining Laboratory at Tianjin University, is one of the most representative large-scale aerial photography datasets available today. It comprises 6471 training images, 548 validation images, and 1610 test images, covering a variety of urban environments such as commercial districts, residential areas, and transportation hubs. VisDrone2019 offers detailed pedestrian annotations, supporting both object detection and tracking tasks. The dataset’s extensive scene variations and diverse target scales provide a solid foundation for enhancing pedestrian detection algorithms’ robustness and generalization capabilities. Additionally, the dataset includes images captured under various weather conditions and different times of day, which aids in improving algorithm adaptability in complex environments. Despite its excellent performance in pedestrian detection, VisDrone2019 has relatively limited data for specific perspectives, such as nadir or vertical angles, which restricts its applicability under extreme conditions. Furthermore, more than the minimal scale variation in pedestrians in the images is needed to support the training of multi-scale pedestrian detection algorithms from UAV perspectives.

The Okutama-Action dataset, collected by Barekatain et al. [20], is primarily intended for human action recognition and detection. This dataset includes 43 min of fully annotated video sequences covering twelve action categories, including running, jumping, and waving. Okutama-Action offers a unique perspective for studying dynamic behaviors by providing pedestrian action data from UAV perspectives. The dataset is valuable for pedestrian behavior analysis, action recognition, and behavior detection in specific environments. However, the pedestrian detection annotations in Okutama-Action are relatively simplistic, and the dataset is mainly focused on the action recognition domain, with limited coverage of diverse scene variations. This results in lower detection accuracy in complex backgrounds. Additionally, the video sequences are relatively short, and the sample size needs to be improved, leading to insufficient generalization capability in large-scale, high-density scenarios.

These datasets offer invaluable data support for pedestrian detection from UAV perspectives, each with its strengths regarding scene coverage, annotation precision, and target variety. Nevertheless, their performance remains limited in various aspects, such as different perspectives, scale variations, and complex backgrounds, and most datasets need more diversity in pedestrian samples. Future research should emphasize addressing pedestrian detection challenges in complex scenarios to advance UAV-based pedestrian detection technologies further. This also provides the contextual foundation for our proposed new dataset, which aims to address the shortcomings of existing datasets by offering more challenging and diverse data, thereby facilitating the research and optimization of pedestrian detection algorithms. In response to the data scarcity and diversity challenges, researchers have proposed a series of solutions based on few-shot learning [21], which holds promise for improving UAV-based pedestrian detection. For instance, Model-Agnostic Meta-Learning (MAML) [22] provides a general meta-learning framework by learning parameter initialization strategies sensitive to new tasks, addressing the few-shot learning problem. Building upon this, Zhang et al. introduced the HelixFormer architecture [23], which models fine-grained semantic relationships between images using a double-helix Transformer structure, further enhancing few-shot learning performance. However, these methods primarily focus on general object detection tasks and have not been sufficiently validated in UAV-based pedestrian detection scenarios, where significant viewpoint variations are a key challenge. This paper is the first to apply few-shot learning strategies to pedestrian detection from UAV perspectives, offering new insights for addressing the data annotation bottleneck in practical applications.

### 2.2. Object Detection

Object detection, a fundamental task in computer vision, has consistently garnered substantial research interest, driving the development of progressively more efficient and accurate detection models. This section provides a comprehensive review of several seminal object detection frameworks that have markedly advanced the field, including Faster R-CNN [24], SSD [25], and the YOLO series [26]. Each of these frameworks has introduced innovative methodologies, significantly enhancing the capabilities and performance of object detection systems.

Faster R-CNN is a widely adopted two-stage object detection framework. It first generates candidate regions using a region proposal network (RPN) and then applies Fast R-CNN [27] for object classification and bounding box refinement. Faster R-CNN demonstrates exceptional detection accuracy and robustness, particularly in handling complex scenes and small objects. However, its high computational complexity presents a bottleneck in real-time processing scenarios. Furthermore, it is optimized for standard perspectives, leading to suboptimal performance when applied to UAV perspectives.

As a single-stage object detection model, SSD performs both detection and classification tasks in a single forward pass. It utilizes multi-scale feature maps for object detection, allowing it to simultaneously identify objects of various sizes while striking an effective balance between accuracy and speed. The computational efficiency of SSD makes it well suited to real-time applications such as autonomous driving and video surveillance. However, SSD relies on predefined anchor boxes, which may result in poor object alignment when applied to UAV perspectives. Additionally, its performance degrades in complex scenes or when detecting small objects, especially compared to two-stage methods.

Since its inception in 2015, the YOLO series has significantly enhanced detection speed and accuracy by integrating detection and classification into a single-stage network. YOLOv3 [28] employs Darknet-53 as its backbone and introduces feature pyramid networks (FPNs) [29] to improve multi-scale object detection capabilities. YOLOv4 [30] further introduced CSPDarknet to enhance feature extraction efficiency while also incorporating a path aggregation network (PAN) [31] to improve the fusion of features across different scales. These innovations have significantly strengthened the feature extraction capabilities and multi-scale object detection accuracy of the YOLO series, enabling it to excel in various complex scenarios. YOLOv7 further boosts feature extraction efficiency and network stability by introducing an extended efficient layer aggregation network (Extended-ELAN) architecture. Its cascading model scaling strategy allows it to adapt to various computational resources and application scenarios, from real-time video surveillance to autonomous driving.

Although these detection methods have laid a solid foundation for computer vision applications, they still face limitations when dealing with UAV perspectives. This highlights the challenge of maintaining detection accuracy and robustness under varying observation conditions while underscoring the necessity of developing detection methods tailored to UAV perspectives. Future research should address these detection challenges in specialized scenarios to advance object detection technologies in broader application domains.

### 2.3. Pedestrian Detection

Current pedestrian detection algorithms can be broadly divided into categories based on handcrafted features and those utilizing deep features. Methods relying on handcrafted features depend heavily on feature engineering and traditional machine learning classifiers. For example, Domonkos et al. proposed the multi-scale center-symmetric local binary pattern (MS-CS-LBP) feature [32], which improves detection performance by effectively capturing local texture information. Ma et al. refined the histogram of oriented gradient (HOG) algorithm [33] and combined it with support vector machines (SVMs) to achieve a robust description of pedestrian contours. To further improve feature representation, Dong et al. integrated Haar-like features with HOG features [34], utilizing an AdaBoost cascade classifier during training, which led to notable improvements in detection performance. However, these approaches need to be more robust in feature representation capacity, making them less adaptable to the complexities of real-world scenarios.

Handcrafted features often have limited expressive power and need help accommodating complex environments’ dynamic variations. To overcome these limitations, researchers have turned their attention to the field of deep learning. He et al. designed a multi-scale detection architecture incorporating anchor-adaptive mechanisms [35], significantly improving detection performance for objects of varying scales. The background-focused distribution alignment (BFDA) framework proposed by Cai et al. markedly enhances detection accuracy through a feature decoupling mechanism [36]. Additionally, Kilicarslan et al. innovatively focused on pedestrian gait characteristics, presenting the Deepstep network [37], which significantly reduces model response time by capturing non-smooth leg movements. Nevertheless, these methods still need to improve robustness when handling small-scale targets and complex occlusion scenarios.

While these approaches have yielded significant results in traditional pedestrian detection tasks, they still demonstrate notable shortcomings when applied to pedestrian detection from UAV perspectives. Challenges such as appearance distortions due to varying viewpoints, increased background interference, and substantial variations in target size hinder the accuracy of feature extraction and target localization, thereby increasing the complexity of the detection process. These limitations suggest that, despite the strong performance of existing pedestrian detection methods in conventional settings, further advancements and innovations are required to address the unique challenges posed by UAV perspectives.

## 3. NSV Dataset

### 3.1. Data Construction

In response to the limitations of existing datasets, including restricted viewpoints, minimal scale variations, simplistic backgrounds, and inadequate sample sizes, this paper presents the Novel Surveillance View (NSV) dataset, an advanced pedestrian detection dataset engineered for UAV perspectives. During the data collection phase, the DJI Mini 3 Pro was utilized as the acquisition platform, equipped with a 1/1.3-inch CMOS image sensor capable of recording 4 K/60 fps video. For this study, data were collected using a 4 K resolution at 30 fps. A systematic multi-dimensional and multi-view collection scheme was developed, spanning a flight altitude range from 10 m to 50 m, with vertical angles varying from 30° to 90°. This three-dimensional collection strategy effectively ensured the completeness and representativeness of data samples across spatial dimensions. Ultimately, 11,566 high-resolution aerial images were gathered, resulting in the construction of the NSV dataset. This dataset provides higher-quality image samples and systematically encompasses key scenarios and challenging viewpoints in UAV-based pedestrian detection for the first time. The specific composition of the NSV dataset is presented in Table 1.

### 3.2. TAHA

Traditional dataset annotation methods predominantly rely on manual operations, which present several limitations. First, this approach’s time-consuming and labor-intensive nature significantly diminishes the efficiency of dataset construction. Second, inconsistencies and accuracy deviations are typical during the annotation process, impacting subsequent algorithms’ training performance. Most critically, when dealing with aerial sequence images, the complexities introduced by variable viewpoints and pronounced differences in target scales further escalate the difficulty and cost of manual annotation.

**Figure 2:**
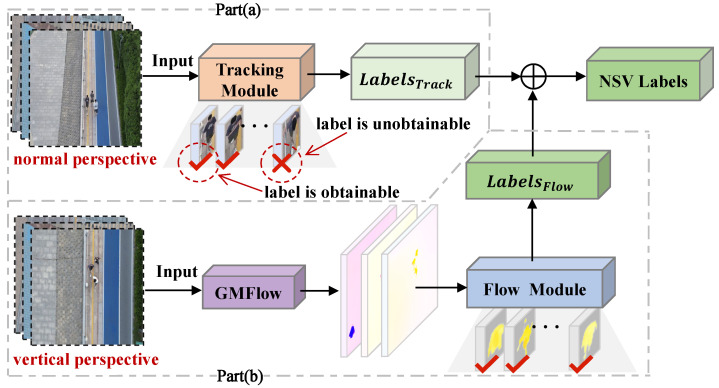
Overall architecture diagram of the TAHA framework. The framework consists of two main modules: (**a**) the target tracking module based on SMILEtrack, which processes target annotations from a normal viewpoint, and (**b**) the optical flow-assisted detection module, designed specifically for target annotations from a top-down viewpoint. The outputs of the two modules (${\mathrm{Labels}}_{\mathrm{Track}}$ and ${\mathrm{Labels}}_{\mathrm{Flow}}$) are fused to achieve robust annotation across different viewpoint scenarios.

To overcome the limitations of traditional manual annotation methods, this study proposes a tracking-based automated hybrid annotation method (TAHA), as illustrated in Figure 2. It effectively integrates annotations from the tracking module under a normal perspective with those from the optical flow module under a vertical perspective. Specifically, in the tracking module, as shown in Figure 2a, we employ a target tracking solution based on SMILEtrack [38]. With its robust tracking capabilities, it effectively handles the rapid motion and morphological changes of targets from a UAV perspective, directly outputting ${\mathrm{Labels}}_{\mathrm{Track}}$. However, during experimental testing, we observed that, as the camera viewpoint gradually transitioned to a top-down angle, the tracking module exhibited an increased likelihood of target loss. This phenomenon mainly arises from significant changes in the posture and appearance features of pedestrians at overhead angles, resulting in a pronounced decline in the performance of conventional tracking models. To address this issue, we innovatively introduce an optical flow-assisted detection module, referred to as the Flow module, as shown in Figure 2b. This module effectively captures annotation information ${\mathrm{Labels}}_{\mathrm{Flow}}$ for pedestrians that conventional tracking methods struggle to acquire from a vertical perspective by analyzing the optical flow of pedestrians. The specific implementation details of the Flow detection module will be elaborated upon in the following subsection. Finally, we conduct manual verification and necessary corrections of the automated annotation results to ensure annotation quality. The TAHA method significantly enhances annotation efficiency and provides consistency and accuracy of annotation quality. Mainly when dealing with top-down scenes, this method effectively overcomes the limitations of traditional target tracking approaches through the assistance of optical flow detection, providing reliable technical support for constructing a high-quality UAV perspective pedestrian dataset.

### 3.3. Flow Module

**Figure 3:**
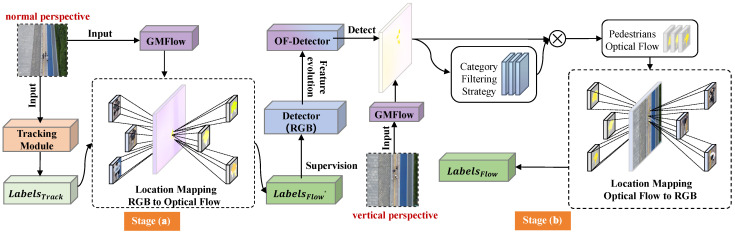
Architecture diagram of the optical flow feature transfer in the Flow module. The framework consists of two key stages: (**a**) the transfer learning stage based on standard perspective data, where the GMFlow algorithm is used to extract optical flow features and feature mapping is performed using detection results from SMILEtrack; (**b**) the vertical perspective detection stage, where a category consistency filtering strategy is applied to achieve accurate detection of pedestrian optical flow features.

This study proposes an automated annotation method based on optical flow feature transfer to address the annotation challenges posed by extreme overhead angles. The core idea of this method stems from an important observation: while the RGB features of targets undergo significant changes at extreme top-down angles, the motion features (optical flow) retain relatively stable expressions. We designed a two-phase annotation framework based on this key finding, as illustrated in Figure 3.

In the first stage, transfer learning is based on normal perspective data. The reason for selecting normal perspective data is that RGB detection can maintain high accuracy at this stage, providing reliable supervisory information for subsequent optical flow feature learning, as illustrated in Figure 3a. Specifically, the GMFlow algorithm [39] is first used to extract the optical flow features from this data, and the reliable detection results obtained from SMILEtrack are precisely mapped to the optical flow feature space. On this basis, a specialized optical flow detector (OF-Detector) is trained for UAV perspectives. In this context, we utilize the state-of-the-art YOLOv7 object detection model to train the detector, ensuring optimal performance and robustness. Then, in the second stage, the trained OF-Detector is applied to extreme overhead angle data, as depicted in Figure 3b. First, the optical flow features of the data are extracted, followed by detection using the OF-Detector. Finally, reverse mapping converts the detection results back to the original image space, resulting in the final annotation information.

**Figure 4:**
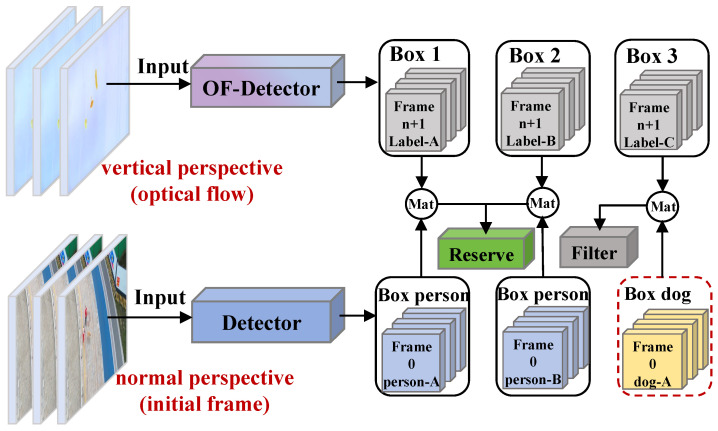
Filtering non-pedestrian labels using initial frames.

The OF-Detector effectively captures the optical flow features of pedestrians. However, in real-world scenarios, other moving objects (such as animals) may lead to false detections, affecting the dataset’s quality. To address this issue, this study innovatively integrates a category-consistency filtering mechanism into the OF-Detector, effectively filtering out non-target optical flow labels by utilizing reliable category information from normal perspectives. This algorithm consists of three core steps: trajectory construction, category validation, and label filtering. First, the optical flow information from video frames taken from a vertical perspective is computed, and the OF-Detector is used to identify moving objects in each frame. For each pair of adjacent frames, let $B_{i}^{t}$ denote the bounding box of the object *i* in frame *t*, and let $IoU(B_{i}^{t},B_{j}^{t+1})$ represent the intersection over the union between bounding boxes in frames *t* and t+1. Our object-matching criterion is defined as follows:

(1) $$IoU\left(B_{i}^{t},B_{j}^{t+1}\right)=\frac{B_{i}^{t}\cap B_{j}^{t+1}}{B_{i}^{t}\cup B_{j}^{t+1}}>\theta .$$

where θ is a predefined threshold. If the overlap ratio exceeds θ, the bounding boxes are considered to belong to the same object. This allows the sequential bounding boxes across frames to be organized into trajectory groups (track boxes). Subsequently, reference trajectory groups are established using the detection results from initial frames, and the unlabeled trajectory groups from the vertical perspective are traced back to the initial frames to establish corresponding relationships with the reference trajectory groups from normal perspectives, accurately obtaining the category identifiers for each trajectory group. Finally, the system automatically filters out trajectories that do not correspond to the pedestrian category, as illustrated in Figure 4. The specific filtering algorithm is presented in Algorithm 1 below.
**Algorithm 1** Category-Consistency Filtering Strategy**Input:** Sequence of video frames *F*, threshold θ
**Output:** Filtered labels with only pedestrian information
  1:Initialize an empty list for object boxes  2:**for** each frame *t* in *F* **do**  3:   Compute optical flow for frame *t*  4:   Detect objects using OF-Detector  5:   **for** each detected object **do**  6:     Calculate bounding box overlap IoU with initial frame  7:     **if** IoU>θ **then**  8:        Assign object to the same box as in the previous frame  9:     **else** 10:        Create a new box for the object 11:     **end if** 12:   **end for** 13:**end for** 14:**for** each box **do** 15:   Trace back to the initial frame using maximum IoU 16:   Identify object category in the initial frame 17:   **if** category == ‘pedestrian’ **then** 18:      Mark as target category 19:   **else** 20:      Mark for removal 21:   **end if** 22:**end for** 23:Remove non-target objects from the dataset 24:**return** Filtered dataset containing only pedestrian labels


This method provides adequate assurance for constructing a high-quality pedestrian detection dataset by fully utilizing multi-perspective information and temporal consistency constraints.

## 4. Pedestrian-DVC Detection Model

**Figure 5:**
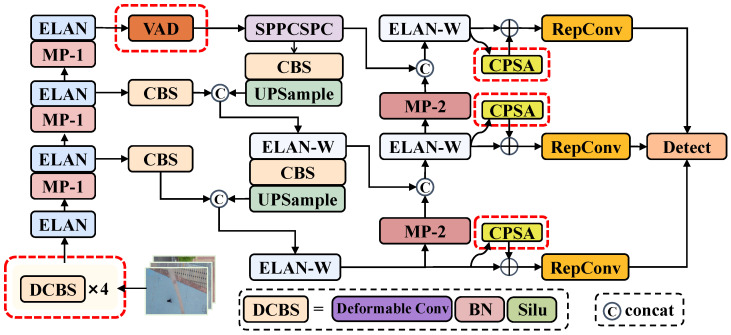
Overall architecture of the Pedestrian-DVC network framework is based on the YOLOv7 architecture and integrates three innovative modules: View-Agnostic Decomposition (VAD) Section 4.1, Deformable Conv-BN-SiLU (DCBS) Section 4.2, and Context-Aware Pyramid Spatial Attention (CPSA) Section 4.3. The innovative modules are highlighted with red dashed circles, and the black dashed boxes represent the specific content or meaning of each module, while the other modules are part of the YOLOv7 architecture.

While mainstream pedestrian detection algorithms have achieved notable success in standard scenarios, they often experience significant performance degradation when confronted with unconventional perspectives, such as those from UAVs. This degradation is especially evident in target recognition and localization accuracy challenges, where existing detectors encounter considerable difficulties. The root cause of this issue lies in the inability of traditional detectors to effectively address the deformations and feature distortions induced by perspective variations, which substantially limits their effectiveness in real-world UAV surveillance applications. To address these challenges, this work proposes Pedestrian-DVC, a pedestrian detection model tailored for UAV perspectives. Based on the YOLOv7 architecture, the model introduces three innovative modules: View-Agnostic Decomposition (VAD), Deformable Conv-BN-SiLU (DCBS), and Context-Aware Pyramid Spatial Attention (CPSA), as shown in the red dashed box in Figure 5.

### 4.1. VAD Module

**Figure 6:**
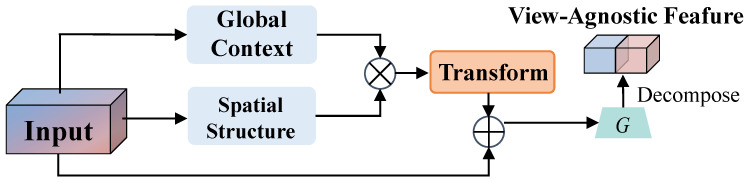
Architecture of the View-Agnostic Detection (VAD) module for extracting stable viewpoint-invariant feature representations.

In practical UAV pedestrian detection scenarios, the complexity of the environment and the unpredictable nature of pedestrian movement trajectories necessitate frequent adjustments of the observation angle to maintain the continuous tracking of targets. This dynamic change in perspective results in significant visual differences of the same pedestrian target in the images, posing challenges related to viewpoint sensitivity for detection models. This paper proposes a VAD module to extract stable viewpoint-invariant feature representations to address this issue, as illustrated in Figure 6.

Given an input feature $F_{in}\in R^{C\times H\times W}$, the VAD module first extracts global context features and spatial structure features through two parallel branches. The global context encoder $E_{g}$ implements a channel attention mechanism through adaptive average pooling and two layers of convolution, capturing global semantic information $F_{g}$. Meanwhile, the spatial structure encoder $E_{s}$ adopts decomposed convolution operations, using 1×3 and 3×1 convolutions to capture horizontal and vertical spatial dependencies, respectively, resulting in $F_{s}$. The corresponding equations are

(2) $$F_{g}=E_{g}\left(F_{in}\right),$$

(3) $$F_{s}=E_{s}\left(F_{in}\right).$$

These complementary features are then fused through an interaction and transformation module $F_{trans}$. The feature transformation module combines pointwise convolution and depthwise separable convolution to enhance the feature representation capability. Finally, a feature selection gating mechanism G(·) adaptively selects the viewpoint-invariant features $F_{out}$, with the detailed equations as follows:

(4) $$F_{trans}=T\left(F_{g}⊗F_{s}\right),$$

(5) $$F_{out}=G\left(F_{in}+F_{trans}\right).$$

The core advantage of this decomposition strategy is that it can effectively filter out feature components affected by viewpoint changes while retaining stable viewpoint-invariant feature representations. The model can maintain consistent detection performance across different viewpoints, significantly improving the detection robustness.

### 4.2. DCBS Module

In the task of pedestrian detection from a UAV perspective, the platform’s maneuverability and dynamic flight altitude often cause pedestrian targets to exhibit irregular geometric shapes and complex posture variations. Due to their fixed sampling patterns, traditional convolution operations struggle to capture these deformable features accurately. To address this limitation, this paper improves the backbone network of the YOLOv7 detection framework by replacing the standard convolution in the CBS (Conv-BN-SiLU) module with a deformable convolution structure, enhancing the model’s geometric adaptability.

Deformable convolution, with its unique adaptive spatial sampling mechanism, can dynamically adjust the shape and size of the receptive field based on the input features. This flexible feature extraction strategy enables the network to more precisely model the non-rigid deformations of pedestrian targets, resulting in more discriminative feature representations. This is particularly advantageous when handling the diversity of pedestrian postures from a UAV perspective. Additionally, due to the unique nature of vertical angles in UAV imagery, pedestrians’ projections can undergo significant viewpoint distortions, which pose substantial challenges for feature extraction. Through its dynamic sampling mechanism, deformable convolution can adaptively adjust the receptive field’s shape according to the target’s actual deformation, enabling a more accurate capture of key pedestrian features. This content-aware adaptive feature extraction mechanism dramatically enhances the model’s robustness to viewpoint variations, allowing the detector to better meet the specific requirements of pedestrian detection from a UAV perspective.

Furthermore, the issue of scale variation in UAV scenarios is effectively addressed. As the flight altitude changes, the size of the same pedestrian target in the image can vary significantly, from tens to hundreds of pixels. Constrained by their fixed receptive field structures, traditional convolution operations need help to handle such large-scale variations. In contrast, deformable convolution learns content-aware sampling offsets, enabling it to effectively adjust the receptive field size to detect pedestrian targets at different scales. This adaptive feature extraction mechanism significantly enhances the model’s scale invariance. These advantages result in improved performance of the modified detector in UAV-based pedestrian detection tasks, especially when dealing with challenging samples characterized by large-scale variations and diverse postures.

### 4.3. CPSA Module

The vertical perspective from UAVs presents a series of unique challenges. Firstly, due to the increased observation distance, the proportion of targets in the image significantly decreases, leading to insufficient feature representation of the targets and amplifying the background interference in feature extraction. Especially in complex urban environments, the visual distinction between background elements such as buildings, vehicles, vegetation, and pedestrian targets is reduced, significantly increasing the difficulty for the detector in feature discrimination. Secondly, UAVs need to adjust their flight altitude dynamically to meet the demands of different scenarios during missions, which directly causes significant scale differences for the same target in the image. Specifically, when the UAV’s flying height changes from tens of meters to hundreds of meters, the size of a pedestrian target may shrink sharply from several hundred pixels to several dozen pixels. This dramatic scale variation poses a severe challenge to the scale adaptability of detectors.

**Figure 7:**
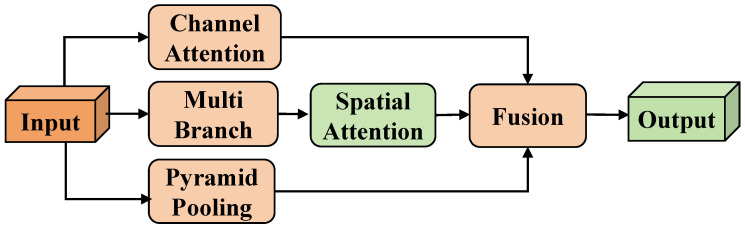
Context-Aware Pyramid Spatial Attention (CPSA) module adaptively processes pedestrian targets at different scales and suppresses background interference.

To effectively address these challenges, this study proposes the Context-Aware Pyramid Spatial Attention (CPSA) module, as illustrated in Figure 7. This module performs feature extraction with different receptive fields through a multi-branch spatial attention structure, utilizing a pyramid pooling mechanism to capture multi-scale contextual information. This design not only adaptively processes features of targets at different scales but also effectively suppresses background interference through global contextual understanding, thereby enhancing the robustness of the detector in complex scenarios.

Specifically, given the input feature $F_{in}^{\prime }$, the CPSA module first performs feature enhancement through multiple branches. The channel attention branch $F_{ca}$ (Channel Attention) recalibrates the importance of channel features through adaptive weights, emphasizing significant channel features while suppressing irrelevant channel responses. The corresponding formula is

(6) $$F_{ca}=\sigma \left(MLP\left(GAP\left(F_{in}^{\prime }\right)\right)\right)⊗F_{in}^{\prime }.$$

where GAP denotes global average pooling, MLP denotes a two-layer perceptron, and σ is the Sigmoid activation function. Next, the multi-branch spatial attention structure $F_{ms}$ (Multi Branch and Spatial Attention) employs three parallel branches $F_{r}$; each branch first reduces the channel dimension through 1×1 convolution to reduce computational overhead, and then achieves multi-scale feature extraction through depthwise separable convolutions with different dilation rates, effectively addressing the dynamic scale changes of targets from the UAV perspective. The specific formulas are

(7) $$F_{r}=Conv_{1\times 1}\left(F_{in}^{\prime }\right),$$

(8) $$F_{ms}=\left\{DWConv\left(F_{r},d_{i}\right)|i=1,2,4\right\}.$$

where $d_{i}$ corresponds to convolution kernels with dilation rates of 1, 2, and 4, with corresponding receptive field sizes of 3×3, 5×5, and 9×9, respectively; DWConv refers to depthwise separable convolution. Then, the pyramid pooling module $F_{pp}$ (Pyramid Pooling) captures hierarchical contextual information from global to local through a multi-scale pooling strategy, aiding in understanding scene structure and suppressing background interference, as given by the following formula:

(9) $$F_{pp}=\left\{Up\left(Conv_{1\times 1}\left(Pool_{k}\left(F_{in}^{\prime }\right)\right)\right)|k\in \left\{1,2,3,6\right\}\right\}.$$

where $Pool_{k}$ denotes an adaptive average pooling operation with an output size of k×k, and Up indicates bilinear interpolation upsampling to the original feature map size.

Finally, the enhanced feature representation is obtained through feature fusion:

(10) $$F_{out}=Fusion\left(Conv\left(F_{ca}\right),Conv\left(F_{sa}\right),Conv\left(F_{pp}\right)\right).$$

where Conv denotes 1×1 convolution for feature alignment, and Fusion performs feature fusion along the channel dimension.

The CPSA module effectively addresses the key challenges the UAV perspective poses through its well-designed, multi-faceted mechanisms. To overcome the limited target feature representation issue, the multi-branch spatial attention structure enhances the model’s ability to capture small targets by extracting features at varying receptive fields. To mitigate the impact of complex background interference, the pyramid pooling module captures hierarchical contextual information, enabling the model to understand scene structure better and distinguish targets from the background. In urban environments, this contextual information allows the model to more accurately differentiate pedestrians from background elements such as buildings and vehicles. Furthermore, the CPSA module enriches the feature representations by employing adaptive feature recalibration, making them more discriminative and significantly boosting detection performance in challenging scenarios. The experimental results demonstrate that this multi-dimensional feature enhancement strategy effectively improves the detector’s robustness, making it better suited to the unique demands of pedestrian detection from a UAV perspective.

## 5. Experiment

### 5.1. Dataset

A notable innovation of the NSV dataset is its highly efficient automated annotation process. Specifically, this study introduces the TAHA method, which enables the automated annotation of large-scale training data. All automatically generated annotated images are selected as dataset frames by choosing the first frame every ten frames in chronological order, thereby ensuring both the size and quality of the dataset. This automated approach not only significantly enhances the efficiency of data annotation but also achieves a groundbreaking expansion in both the scale and diversity of the dataset.

From a data distribution perspective, the NSV dataset exhibits substantial diversity across several dimensions: (a) viewpoint variations, (b) target-to-camera distances, (c) lighting conditions, and (d) background complexity. This multi-dimensional variability introduces new challenges to pedestrian detection tasks while providing essential support for enhancing the model’s generalization capabilities. To ensure the reliability of evaluations, the test set employs independent scenes and meticulous manual annotation strategies. The independent scene setup allows a more objective assessment of the model’s generalization performance. At the same time, high-quality manual annotations provide a reliable benchmark for model performance evaluation and validate the effectiveness of the semi-automated annotation method employed in the training set.

This study compares the NSV dataset with the VisDrone2019 and Okutama-Action datasets, with specific information detailed in Table 2.

As can be observed from the table presented above, the NSV dataset demonstrates significant advantages over the VisDrone2019 and Okutama-Action datasets regarding viewpoint diversity, scale diversity, and sample diversity. Specifically, the NSV dataset encompasses a broader range of camera viewpoints, including various heights and angles, making pedestrian detection more challenging across different perspectives. Regarding scale diversity, the pedestrian targets in the NSV dataset span a wide range of distances, from far to near, enhancing the model’s ability to adapt to targets of varying sizes. Additionally, the increased sample diversity means that the dataset includes a greater variety of pedestrian postures, attire, and dynamic behaviors, further enriching the diversity of training samples. This multi-dimensional advantage introduces more complex and realistic scene challenges to pedestrian detection tasks and provides a robust foundation for improving the model’s generalization capabilities. By training in a diversified data environment, the model can more effectively handle complex real-world application situations, maintain high detection performance, and significantly enhance its practicality and reliability in actual scenarios.

### 5.2. Evaluation Metrics and Experimental Setup

To thoroughly evaluate the performance of the improved pedestrian detection model under UAV perspectives, this study utilizes several standard evaluation metrics, including Average Precision (AP), Precision, and Recall, to assess the model’s accuracy and robustness across different aspects. Specifically, AP@0.5 represents the average precision with an IoU threshold of 0.5, while AP@0.5:0.95 refers to the average precision computed over IoU thresholds ranging from 0.5 to 0.95, with a step size of 0.05. The formula for precision is given as follows:

(11) $$Precision=\frac{TP}{TP+FP}.$$

where TP (True Positive) is the number of true positives and FP (False Positive) is the number of false positives. The formula for Recall is expressed as follows:

(12) $$Recall=\frac{TP}{TP+FN}.$$

where FN (False Negative) is the number of false negatives. The formula for AP@0.5:0.95 is

(13) $$AP_{0.5:0.95}=\frac{1}{10}\sum_{i=0}^{9}AP_{IoU=0.5+0.05i}.$$

These evaluation metrics comprehensively assess the pedestrian detection model’s performance under different scenarios and conditions, offering references for subsequent improvements and optimizations.

Regarding the loss function, a combination of cross-entropy loss and IoU loss is utilized to improve the model’s detection accuracy and localization precision. The $L_{CE}$ (cross-entropy loss) measures the difference between predicted and true class labels, expressed as follows:

(14) $$L_{CE}=-\sum_{i=1}^{N}y_{i}\log\left(p_{i}\right).$$

where $y_{i}$ is the true class label, $p_{i}$ is the predicted probability, and *N* is the number of samples. The IoU loss evaluates the overlap between the predicted bounding box and the ground truth, represented as follows:

(15) $$L_{IoU}=1-\frac{I(A,B)}{U(A,B)}.$$

where *A* is the predicted bounding box, *B* is the ground truth bounding box, I(A,B) denotes the intersection area of the predicted and ground truth boxes, and U(A,B) represents the union area.

The combined loss function, which incorporates both $L_{CE}$ and $L_{IoU}$, is expressed as follows:

(16) $$L=\alpha \cdot L_{CE}+\beta \cdot L_{IoU}.$$

where α and β are weight parameters used to balance classification loss and localization loss. In the experiments, these parameters are set to 0.05 and 0.2, respectively, to emphasize accurate pedestrian localization while maintaining classification capability for a single category.

The experiments in this study were conducted using the COCO-YOLOv7 model as the baseline and trained on the NSV dataset. All experiments were executed on a single NVIDIA RTX 4090 GPU, within the PyTorch 1.13.1 framework utilizing CUDA 11.6. The experimental environment included Python 3.9.7, along with libraries such as NumPy 1.21.5, OpenCV 4.5.5, and Albumentations 1.3.0 for preprocessing and data augmentation.

Preprocessing operations included image scaling, normalization, and data augmentation techniques such as random cropping, flipping, and brightness adjustment, aimed at improving the model’s generalization ability. For training, the AdamW optimizer was used, combining adaptive learning rates with weight decay. The initial learning rate was set to 0.001, and a cosine annealing scheduler was employed to gradually reduce it, ensuring stable convergence. The weight decay coefficient was set to 0.0005 to prevent overfitting. The batch size was set to 16 to fully leverage GPU resources. The model was trained for 100 epochs, with performance metrics, including loss values, recorded at each epoch. To ensure reproducibility, random seeds were fixed across all experiments.

### 5.3. Ablation Experiment

This study conducted ablation experiments to verify the contributions of the DCBS, VAD, and CPSA modules in enhancing pedestrian detection performance from a UAV perspective by incrementally adding key modules on the NSV test set. The results of the ablation experiments are presented in Table 3.

The table’s data clearly demonstrate that each proposed enhancement module significantly improves detection performance, with the VAD module contributing the most noticeable gains. Specifically, introducing the VAD module resulted in a 5.1% increase in AP@0.5 compared to the baseline model. This substantial improvement can be attributed to the innovative design of the VAD module in feature extraction, which effectively decomposes and preserves stable, perspective-invariant feature representations. Notably, the VAD module efficiently filters out perspective-induced interference features when addressing dynamic changes in UAV perspectives, ensuring consistent performance across varying observation angles.

Building upon the VAD module, the addition of the CPSA module further improved model performance by 2.5%. This enhancement can be attributed to the multi-scale feature refinement mechanism of the CPSA module. Its multi-branch spatial attention structure, leveraging depthwise separable convolutions with varying dilation rates, increases the model’s adaptability to targets of different scales. Secondly, the hierarchical contextual information the pyramid pooling module provides significantly enhances the model’s ability to interpret complex backgrounds. This composite feature enhancement strategy equips the detector with greater robustness, enabling it to detect targets at varying flight altitudes more effectively.

Finally, the DCBS module, which incorporates deformable convolutions, is introduced, enhancing the model’s average precision, accuracy, and recall. This improvement stems from the ability of deformable convolutions to overcome the limitations of traditional convolutions with fixed receptive fields, allowing for dynamic adjustment of sampling positions based on the actual deformation of the target. This adaptive feature extraction mechanism is particularly effective in handling the irregular geometric deformations of pedestrian targets from UAV perspectives, enabling the model to capture key features of the targets more accurately.

In summary, the synergistic integration of these three modules forms a comprehensive feature enhancement framework: the VAD module ensures perspective invariance, the CPSA module enhances multi-scale feature representation, and the DCBS module offers more flexible feature sampling capabilities. While this multi-layered design substantially boosts detection accuracy in UAV-based pedestrian scenarios, it also introduces additional parameters and computational overhead, resulting in a slight reduction in FPS. Nevertheless, for most UAV applications, the benefits in detection performance justify these overheads, making the proposed model a viable solution for real-world deployment.

### 5.4. Comparison Experiments

To effectively demonstrate the efficacy of the proposed TAHA method, we conducted comparative experiments to evaluate the impact of different tracking methods and optical flow estimation techniques on the dataset and experimental outcomes. Table 4 presents the detailed experimental results.

The table above shows that the proposed TAHA method exhibits stable performance across various tracking methods and optical flow estimation techniques, further validating its effectiveness. This approach significantly reduces the labeling workload for researchers and paves the way for future advancements in automated dataset labeling.

Additionally, to better demonstrate the effectiveness of our model for pedestrian detection from a UAV perspective, this study compares its performance against various object detection models and state-of-the-art pedestrian detection models on the NSV dataset. All models in the comparative experiments use standard weights pre-trained on the COCO dataset. Table 5 presents the experimental results for the different models.

As shown in the table above, the improved model achieves AP@0.5 improvements of 17.2% and 15.6% over SSD and Faster R-CNN, respectively. Compared to the YOLO series models—YOLOv3, YOLOv5, YOLOv7, YOLOv8, and YOLOv11—the improvements are 14.3%, 13.1%, 9%, 9.4%, and 7.6%, respectively. Additionally, it outperforms the pedestrian detection models ACSP, Cascade R-CNN, and F2DNet by 13.7%, 12.9%, and 11%, respectively. Considering both accuracy and model complexity, these results demonstrate the clear advantages of the YOLO series for pedestrian detection from a UAV perspective.

### 5.5. Generalization Comparison Experiments

To further validate the reliability and generalization of the proposed method, we performed additional tests on external benchmarks beyond the specifically constructed NSV dataset. These external datasets include the widely used VisDrone2019 and Okutama-Action datasets. For the VisDrone2019 and Okutama-Action datasets, we carefully selected only the pedestrian-related data that align with the UAV perspective to ensure the relevance and accuracy of the experimental results. The improved model was then systematically compared with existing object and pedestrian detection models to evaluate its performance across various datasets. The detailed experimental results are presented in Table 6.

Our experimental analysis demonstrates that the proposed model not only achieves strong performance on the custom NSV dataset but also excels in pedestrian detection on well-established public datasets like VisDrone2019 and Okutama-Action. These results highlight the robustness and effectiveness of the proposed approach, reinforcing its practical applicability in a wide range of real-world UAV vision scenarios. The model’s success on these diverse benchmarks further emphasizes its potential for generalization across different environmental conditions and camera perspectives.

### 5.6. Few-Shot Learning

In practical applications, obtaining large-scale annotated datasets is often constrained by resource and time limitations, particularly in UAV-based pedestrian detection tasks. These tasks require capturing diverse viewpoints and accurately annotating images, making the entire process both time-consuming and labor-intensive. To address this challenge, this study explores the potential of few-shot learning strategies in extreme viewpoint pedestrian detection. Specifically, we first pre-train the model on the VisDrone2019 dataset to acquire general feature representation capabilities, and then fine-tune the model using a small number of samples from the NSV dataset to assess its rapid adaptability under extreme top-down perspectives. In the experimental setup, we specifically selected scene samples from the NSV dataset with viewpoints greater than 80 degrees and constructed two evaluation scenarios: 1-shot and 5-shot. The 1-shot scenario uses only one annotated sample per scene for fine-tuning, while the 5-shot scenario uses five annotated samples. To ensure the reliability of the experiments, we randomly selected the support set (used for fine-tuning) and query set (used for testing), and conducted multiple repeated experiments to obtain an average result. The specific experimental results are shown in Table 7.

The experimental results demonstrate that, even with extremely limited annotated data, the proposed method still exhibits strong transfer learning capabilities. Compared to traditional fine-tuning methods, the model achieves an AP@0.5 of 61.9% with just one sample (1-shot), which is a 2.1% improvement over the baseline. When the number of samples is increased to five (5-shot), the performance further improves to 65.6%, showing a significant performance gain. These results validate the practical value of the proposed method, particularly its ability for rapid deployment in scenarios with limited annotation resources.

### 5.7. Visualization Analysis

**Figure 8:**
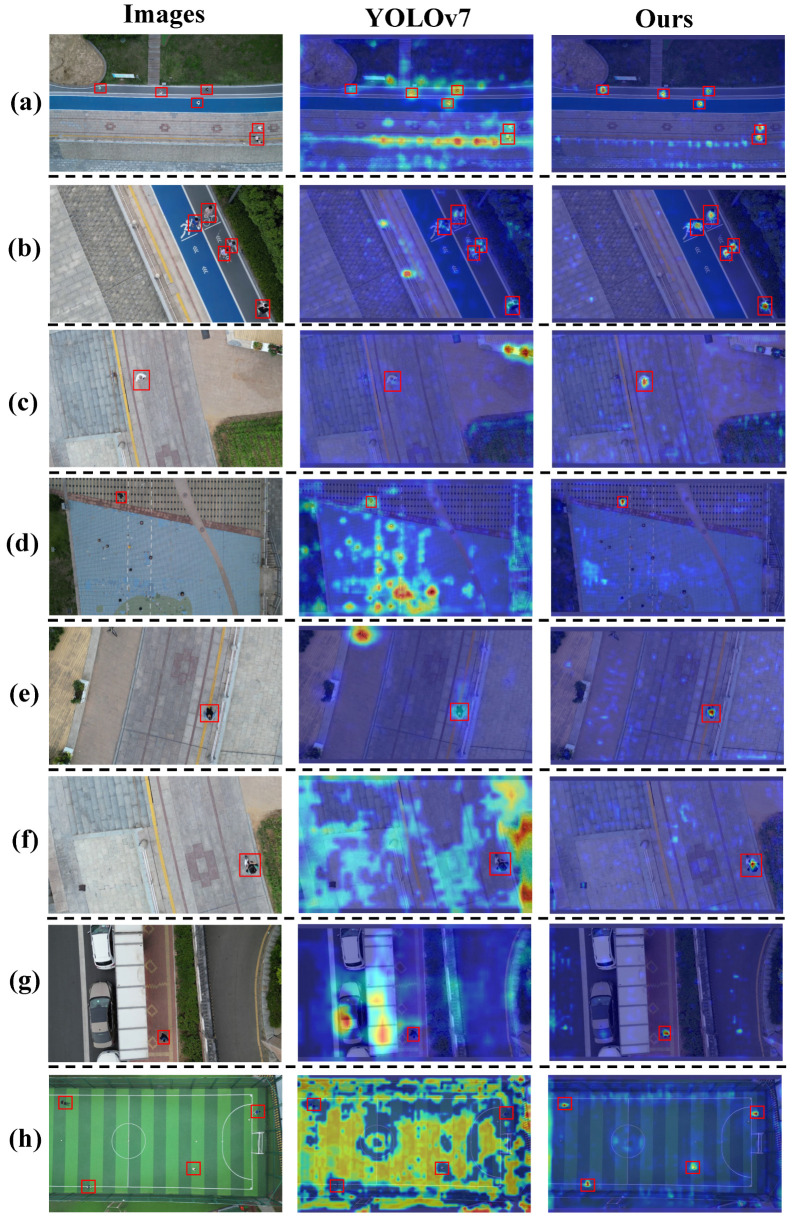
Comparison of visualization information of attention heatmaps. (**a**–**h**) Each group shows the attention heatmap results for the same image using different methods. In each heatmap, areas with redder tones indicate higher levels of attention. Red boxes denote the ground truth locations of pedestrians.

To intuitively demonstrate the model’s performance and improvements in pedestrian detection from a UAV perspective, we visualized several detection results and heatmap data from the test set. This visualization highlights the model’s detection capabilities in various complex scenarios, including variations in pedestrian orientation, scale, and environmental lighting conditions. The heatmap results from the improved model on the test set are shown in Figure 8.

**Figure 9:**
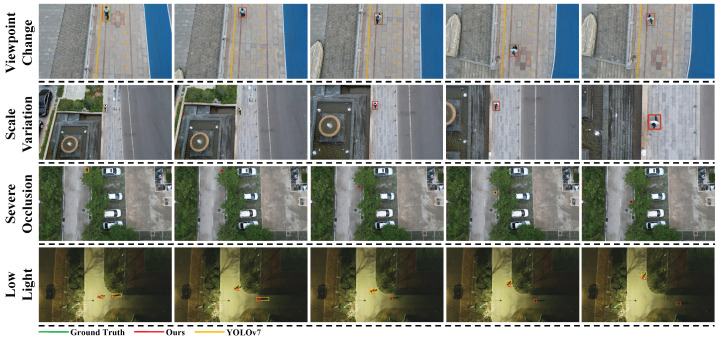
Performance of the Pedestrian-DVC model on the NSV testset.

Through visual analysis of the attention heatmaps, we can observe the model’s attention distribution patterns for pedestrian targets from a UAV perspective. The experimental results demonstrate a significant improvement in the feature extraction capability of the Pedestrian-DVC model. High-response features are prominently concentrated in the pedestrian target regions, while background areas show low response values. The model maintains a stable and focused attention distribution even when the target size is small. Additionally, the detection performance of the improved model on the test set is illustrated in Figure 9.

The Pedestrian-DVC model demonstrates robust pedestrian detection performance across various challenging scenarios, as highlighted by the visualizations in Figure 9, where we show comparisons between the original YOLOv7 and our model under different conditions. Specifically, the model can accurately detect pedestrians with varying postures, scales, and distances. It maintains high detection accuracy even at more vertical overhead angles, where traditional models struggle, successfully identifying the majority of pedestrians. In addition, the model performs well in scenes with suboptimal lighting conditions, such as low light, where the YOLOv7 model fails to detect pedestrians reliably. Furthermore, it effectively handles complex backgrounds and severe occlusions, making it more robust in real-world scenarios where pedestrians are often partially obscured by obstacles or other objects. The model also adapts well to scale variations, detecting pedestrians at various sizes with high accuracy. This is particularly evident in the multi-scale visualizations, where the model outperforms YOLOv7 in detecting pedestrians at different distances from the UAV. These results demonstrate the model’s advanced feature extraction capabilities, distinguishing pedestrians from the background even in challenging conditions like viewpoint changes, scale variations, low lighting, and severe occlusions, thereby showcasing its robustness and generalization ability.

## 6. Discussion

Experimental results on our NSV and public datasets VisDrone2019 and Okutama-Action demonstrate that Pedestrian-DVC outperforms existing algorithms. However, the experiments also reveal limitations, such as the relatively large model weights, which lead to significant resource consumption when deployed on UAVs. Future work will focus on fine-tuning hyperparameters, refining the network architecture, and addressing these resource-related issues.

In addition, the current NSV dataset primarily focuses on RGB imagery and lacks multimodal information, such as infrared (IR) and depth data. These modalities are particularly valuable for addressing challenges such as low-light conditions, complex backgrounds, and occlusions, which are prevalent in UAV-based pedestrian detection scenarios. As part of future work, we plan to enhance the NSV dataset by incorporating synchronized RGB, IR, and depth data. To achieve this, we intend to use DJI’s newly released M4T UAV as the foundational platform for multimodal data collection. This advanced UAV platform is equipped with integrated RGB-IR cameras and supports the addition of depth sensors (e.g., stereo vision or LiDAR), enabling the efficient collection of synchronized multimodal data under diverse conditions.

The expanded NSV dataset will better represent UAV surveillance scenarios across varying times of day, weather conditions, and flight altitudes. The inclusion of infrared and depth modalities will also facilitate the exploration of multimodal fusion techniques, leveraging the complementary strengths of different data sources to enhance the detection robustness. For instance, infrared imaging can improve detection in low-visibility environments, while depth data provide spatial information to help distinguish pedestrians from complex backgrounds. By integrating these modalities and leveraging the capabilities of cutting-edge UAV platforms like the DJI M4T, the NSV dataset aims to become a more holistic benchmark, promoting further advancements in pedestrian detection from UAV perspectives.

## 7. Conclusions

This paper presents a novel pedestrian detection method for UAVs based on YOLOv7, addressing the significant performance degradation observed in traditional detectors when applied to overhead views. First, we introduced a new pedestrian detection dataset tailored for UAV applications, employing an automated annotation strategy that significantly improved data acquisition efficiency while maintaining high annotation quality. This dataset offers higher-quality image samples and systematically covers key scenarios and challenging perspectives in UAV-based pedestrian detection for the first time. Second, we designed the Pedestrian-DVC detection framework, incorporating three innovative modules built upon the YOLOv7 architecture. The DCBS module adapts the receptive field using deformable convolutions to effectively handle the geometric distortions of pedestrian appearances from the UAV perspective. The VAD module decouples pedestrian features into angle-dependent and angle-independent branches, significantly enhancing the model’s robustness against viewpoint variations and improving its generalization across different observation angles. The CPSA module leverages a multi-scale feature pyramid structure combined with a spatial attention mechanism to effectively tackle the problem of severe target scale variations in UAV-based detection.

The experimental results demonstrate that the proposed Pedestrian-DVC framework outperforms the baseline model across all evaluation metrics, effectively addressing the unique challenges of pedestrian detection from a UAV perspective. This work provides a new research direction and offers a practical solution for pedestrian detection tasks from UAV perspectives. 

## Figures and Tables

**Table 1:** Composition of the NSV dataset.

Scene Type	Quantity	Time of Day	Weather Conditions	Altitude Range	View Angle Range	Pedestrian Density
Square	20%	Day and night	Sunny and cloudy	15–50 m	30–90°	Sparse and dense
Park	20%	Day and night	Sunny and cloudy	15–50 m	30–90°	Sparse and dense
Seaside	20%	Day and night	Sunny and cloudy	15–50 m	30–90°	Sparse and dense
Campus	20%	Day and night	Sunny and cloudy	15–50 m	30–90°	Sparse and dense
Urban Streets	10%	Day and night	Sunny and cloudy	10–30 m	30–90°	Sparse and dense
Bus Station	5%	Day	Sunny and cloudy	10–15 m	30–90°	Sparse and dense
Parking Lot	5%	Day	Sunny and cloudy	10–15 m	30–90°	Sparse

**Table 2:** Detailed information on the various datasets.

Name	Type	Format	Number	Perspective	Scale
VisDrone2019	Training		6471		
	Validation	Image	548	Fewer	Moderate
	Testing		1610		
Okutama-Action	Training		33		
	Validation	Video	None	Moderate	Fewer
	Testing		10		
NSV	Training		8852		
	Validation	Image	2214	More	More
	Testing		500		

**Table 3:** Ablation experiment.

Method	VAD	CPSA	DCBS	AP@0.5	AP@0.5:0.95	Precision	Recall	Params (M)	Flops (G)	FPS
1				59.8	34.5	61.7	53.5	36.4	103.2	89
2	✓*			64.9	38.5	68.3	59.6	37.1	105.1	88.6
3	✓	✓		67.4	39.3	70.4	62.4	38.6	108.4	86.5
Ours	✓	✓	✓	68.8	40.1	70.9	69.3	41.5	113.6	83.2

* Checkmark indicates the inclusion of this module.

**Table 4:** Comparison experiment of different models in TAHA.

ByteTrack [40]	SMILEtrack	FlowFormer [41]	GMFlow	NSV Quantity	AP@0.5	AP@0.5:0.95
✓*		✓		11,478	68.4	39.7
✓			✓	11,485	68.6	39.8
	✓	✓		11,574	68.8	39.9
	✓		✓	11,566	68.8	40.1

* Checkmark indicates the inclusion of this method.

**Table 5:** Comparison experiment of different models.

Model	AP@0.5	AP@0.5:0.95	Model Weight (MB)
SSD	51.6	29.4	159
Faster R-CNN	53.2	30.8	135
YOLOv3	54.5	31.7	236
ACSP [42]	55.1	32.3	321
YOLOv5	55.7	33.1	166
Cascade RCNN [43]	55.9	33.9	315
F2DNet [44]	57.8	34.2	217
YOLOv8	59.4	34.8	50
YOLOv7	59.8	34.5	72
YOLOv11 [45]	61.2	34.4	39
Ours	68.8	40.1	95

**Table 6:** Validation results on public datasets.

Model	Testing	AP@0.5	AP@0.5:0.95
SSD	VisDrone2019	39.7	26.3
Faster R-CNN	VisDrone2019	40.9	26.8
ACSP	VisDrone2019	41.9	27.4
YOLOv3	VisDrone2019	42.8	28.1
YOLOv5	VisDrone2019	43.7	28.6
Cascade RCNN	VisDrone2019	44.6	29.1
F2DNet	VisDrone2019	45.5	29.7
YOLOv7	VisDrone2019	46.4	30.2
YOLOv8	VisDrone2019	46.7	30.1
YOLOv11	VisDrone2019	46.9	30.5
Ours	VisDrone2019	50.2	32.9
SSD	Okutama-Action	54.6	31.2
Faster R-CNN	Okutama-Action	56.2	32.3
YOLOv3	Okutama-Action	56.7	32.9
YOLOv5	Okutama-Action	58.4	33.5
ACSP	Okutama-Action	59.1	34.3
Cascade RCNN	Okutama-Action	60.2	35.4
F2DNet	Okutama-Action	61.5	35.7
YOLOv8	Okutama-Action	63.3	35.5
YOLOv7	Okutama-Action	63.6	36.1
YOLOv11	Okutama-Action	63.8	35.9
Ours	Okutama-Action	69.2	38.7

**Table 7:** Few-shot learning result.

Method	Scenario	AP@0.5	AP@0.5:0.95	Precision	Recall
Baseline	Full	59.8	34.5	61.7	53.5
Ours		68.8	40.1	70.9	69.3
Fine-tuning	1-Shot	60.6	35.6	62.9	61.5
MAML		61.9	36.3	64.1	62.1
Fine-tuning	5-Shot	62.3	36.4	64.3	62.9
MAML		65.6	38.2	67.7	66.1

## Data Availability

The Novel Surveillance View (NSV) dataset is now publicly available at https://github.com/gaoshengran/NSV-dataset (accessed on 15 December 2024).
